# PP11 Evaluating Environmental Sustainability In Health Technology Assessment: A Multisectoral Systematic Review

**DOI:** 10.1017/S0266462324001843

**Published:** 2025-01-07

**Authors:** Melissa Pegg, Janet Bouttell, Matthew Taylor

## Abstract

**Introduction:**

Research widely reports healthcare technologies contribute substantial environmental impacts. Timely health technology assessment (HTA) environmental sustainability (ES) framework development is critical. A lack of multisectoral expertise, resource constraints, and consensus to assess environmental impact is delaying methodological progression. The study objective was to identify and critically evaluate methods supporting ES in HTA and formulate recommendations to underpin the development of an ES framework in HTA.

**Methods:**

This multisectoral systematic review followed PRISMA guidelines. Maximizing the opportunity to evaluate applied environmental assessment methods, the databases Web of Science, Embase, PubMed, IEEE Xplore, EBSCOhost GreenFILE, Cochrane Library, and International Network of Agencies for Health Technology Assessment (INAHTA) were searched between 2008 and 2023. Full-text published studies applying both qualitative and quantitative methods to evaluate technology ES were included. Frameworks were critiqued for their comprehensiveness based on sustainability scope and in challenging barriers to decision-making. Studies were ranked according to how transparent and feasible frameworks were in their ability to assess technology ES and alignment with HTA principles.

**Results:**

A total of 10 studies were identified and ranked in order of suitability in assessing technology ES in HTA. All studies applied a combination of methods to overcome issues such as data and resource constraints, expert knowledge, consensus in decision-making, and multiple criteria trade-off. Ranked highest, the One Health “extended” life cycle assessment (LCA) framework that utilized SimaPro LCA advanced software addressed the greatest methodological needs for HTA. Ranked second, the circular economy (CE) framework used in conjunction with an analytical hierarchy process (AHP) makes use of weighting and expert consultation to support multiple-criteria decision-making (MCDM).

**Conclusions:**

This is the first systematic review to identify multidisciplinary frameworks, supporting evaluation of ES and decision-making in HTA. Providing valuable insight into potential methodological solutions where there is limited research, this study facilitates ES policy development in HTA. This study challenges limitations to methodological development highlighted by previous research. Further research should apply these recommendations in an HTA setting.

